# Associative learning of classical conditioning as an emergent property of spatially extended spiking neural circuits with synaptic plasticity

**DOI:** 10.3389/fncom.2014.00079

**Published:** 2014-07-25

**Authors:** John H. C. Palmer, Pulin Gong

**Affiliations:** ^1^School of Physics, University of SydneySydney, NSW, Australia; ^2^Sydney Medical School, University of SydneySydney, NSW, Australia

**Keywords:** associative learning, STDP, STD, propagating waves, spike sequences, classical conditioning

## Abstract

Associative learning of temporally disparate events is of fundamental importance for perceptual and cognitive functions. Previous studies of the neural mechanisms of such association have been mainly focused on individual neurons or synapses, often with an assumption that there is persistent neural firing activity that decays slowly. However, experimental evidence supporting such firing activity for associative learning is still inconclusive. Here we present a novel, alternative account of associative learning in the context of classical conditioning, demonstrating that it is an emergent property of a spatially extended, spiking neural circuit with spike-timing dependent plasticity and short term synaptic depression. We show that both the conditioned and unconditioned stimuli can be represented by spike sequences which are produced by wave patterns propagating through the network, and that the interactions of these sequences are timing-dependent. After training, the occurrence of the sequence encoding the conditioned stimulus (CS) naturally regenerates that encoding the unconditioned stimulus (US), therefore resulting in association between them. Such associative learning based on interactions of spike sequences can happen even when the timescale of their separation is significantly larger than that of individual neurons. In particular, our network model is able to account for the temporal contiguity property of classical conditioning, as observed in behavioral studies. We further show that this emergent associative learning in our network model is quite robust to noise perturbations. Our results therefore demonstrate that associative learning of temporally disparate events can happen in a distributed way at the level of neural circuits.

## Introduction

Associating sequential events happening at different time moments is of fundamental importance for a host of perceptual and cognitive functions (Wallenstein et al., 1998; Fuster et al., 2000). One of the important paradigms of such association is classical conditioning, in which the pairing of two subsequent stimuli is learned such that the presentation of the CS is taken as a predictor of the subsequent US (Rescorla, 1988). Despite the fact that all classical conditioning experiments have an important temporal component (i.e., the US occurs after the CS), many theories such as the Rescorla-Wagner theory of conditioning do not include this timing relationship in their models (Rescorla and Wagner, 1972; Markram et al., 2011). Furthermore, most of the theoretical models proposed for classical conditioning have focused on individual neurons or synapses by assuming the presence of slowly decaying firing activity of neurons. This, when combined with some synaptic learning rules such as spike timing dependent plasticity (STDP), enables associative learning between temporally separated events to happen (Gluck and Thompson, 1987; Wörgötter and Porr, 2005; Drew and Abbott, 2006). However, experimental evidence for such slowly decaying firing activity is still inconclusive (Ito et al., 2008).

Here, we present a novel, alternative account of associative learning of spatially and temporally separated events, which utilizes the emergent dynamics of neural circuits rather than individual neurons. Our proposal relies on two basic, known neurophysiological features: (1) spike-timing dependent plasticity (Abbott and Nelson, 2000; Dan and Poo, 2006), and (2) short term depression (STD) (Zucker and Regehr, 2002). In our proposal, both the CS and US are encoded as spike sequences formed by propagating spiking waves, which emerge from spatially extended, spiking neural circuits, in which each neuron is coupled to nearby neurons in two dimensions, with coupling strengths being a function of distance. Associating the CS and the US with a time separation greater than 100 ms naturally occurs through the timing-dependent interacting dynamics of these spiking waves. We further show that our model of associative learning is able to account for the temporal contiguity property of classical conditioning, i.e., that the success of associative learning of conditioning is a non-monotonic function of the amount of time by which the CS precedes the US; in particular when the precedence is either too short or too long, conditioning is relatively poor, as found in behavioral studies (Rescorla, 1988). We also show that our model is robust against perturbations.

The idea of using interacting waves to account for the associative learning of different stimuli can be traced back to Beurle's landmark study (Beurle, 1956). Since this work, however, studies of association have focused on associatively recalling a stimulus based on partial clues, mainly dominated by the view of fixed point attractors (Hopfield, 1982). Recently, evidence of propagating waves in neural systems has been rapidly accumulating (Rubino et al., 2006; Benucci et al., 2007; Ferezou et al., 2007; Han et al., 2008; Wu et al., 2008; Lubenov and Siapas, 2009; Sato et al., 2012), strongly suggesting that it is time to consider the fundamental question regarding the potential functional roles of propagating waves in the brain (Gong and Van Leeuwen, 2009). Our new model of associative learning is a step forward along this direction.

## Materials and methods

### Neural circuit model

We consider a *n*-by-*n* two dimensional lattice of integrate-and-fire (IF) neurons. We denote the membrane potential of a neuron at integer coordinates (*i, j*) at time *t* by *V_ij_(t)*, which has dynamics governed by the following equation,

(1) $$V_{ij}\left(t+\Delta t\right)=\{\begin{array}{ll} e^{-\Delta t/\tau }V_{ij}\left(t\right)+V^{ex}+V_{ij}^{in}(t) & \text{if }V\left(t\right)<V_{th},\\ V_{ij}\left(t\right)-V_{th} & \text{if }V\left(t\right)\geq V_{th}, \end{array}$$

where τ = 20 ms is the neural time constant, Δ*t* = 1 ms is the time step, *V*^*ex*^ is a constant external input, *V*^*in*^_*ij*_ (*t*) is the input received from other neurons at time *t*. Here both the constant external input and the input due to the spiking neurons both have an implicit dependence on Δ*t*. However, as Δ*t* is constant, this dependence is absorbed into the appropriate term. A spike is generated whenever the neuron reaches a threshold voltage, *V*_*th*_ = 1. The coupling strength between any two neurons located at (*i, j*) and (*i′, j′*) respectively is denoted as *W_ij,i'j'_* constructed from a “Mexican-hat” function:

(2) $$W_{ij,i^{\prime }j^{\prime }}^{\prime }=\{\begin{array}{ll} C_{E}e^{-d_{ij,i^{\prime }j^{\prime }}^{2}/d_{E}^{2}}-C_{I}e^{-d_{ij,i^{\prime }j^{\prime }}^{2}/d_{I}^{2}} & \text{if }d_{ij,i\prime j\prime }\leq D_{1},\\ 0 & \text{if }d_{ij,i\prime j\prime }>D_{1} \end{array}$$

where *d_ij_,i′j′* is the Euclidean distance between neurons on a lattice with periodic boundary conditions, *C*_*E*_ = 0.4, *C*_*I*_ = 0.1, $d_{E}=\sqrt{14},d_{I}=\sqrt{42}$ are constants that determine the shape of the coupling function. As interactions between neurons in real neural systems have finite ranges, connections between neurons in the model are constrained to *d_ij,i′j′_* < *D*_1_ = 15. For these parameter choices, coupling is excitatory if $d_{ij,i^{\prime }j^{\prime }}<D_{0}=\sqrt{21\log(4)}$ and inhibitory if *D*_1_ > *d_ij,i′j′_* > *D*_0_, therefore resulting in a lateral inhibitory coupling structure; such lateral inhibition could be achieved through disynaptic pathways (Melchitzky et al., 2001) or large basket cells (Markram et al., 2004). Such spiking neural circuits with lateral inhibition have been commonly used to model neural systems including the hippocampus (Samsonovich and Mcnaughton, 1997). Note that we have found that the collective dynamics such as the formation of spiking waves in the neural circuit are not sensitive to the values of any of the above parameters, as shown in Palmer and Gong (2013).

To enable the total excitatory and inhibitory coupling strengths to be changed without varying their spatial extent, they are normalized separately using the following normalization constants:

(3) $$\begin{array}{l} W_{ij}^{E}=\sum_{i^{\prime }j^{\prime }}W_{ij,i^{\prime }j^{\prime }}^{\prime }\text{ if }d_{ij,i^{\prime }j^{\prime }}\leq D_{0},\\ W_{ij}^{E}=\sum_{i^{\prime }j^{\prime }}W_{ij,i^{\prime }j^{\prime }}^{\prime }\text{ if }D_{0}<d_{ij,i^{\prime }j^{\prime }}<D_{1}. \end{array}$$

The coupling strengths are then given by

(4) $$W_{ij,i^{\prime }j^{\prime }}=\{\begin{array}{ll} \frac{W_{E}W_{ij,i^{\prime }j^{\prime }}^{\prime }}{W_{ij}^{E}} & \text{ if }d_{ij,i^{\prime }j^{\prime }}<D_{0},\\ \frac{W_{I}W_{ij,i^{\prime }j^{\prime }}^{\prime }}{W_{ij}^{I}} & \text{ if }D_{0}<d_{ij,i^{\prime }j^{\prime }}<D_{1},\\ 0 & \text{ if }d_{ij,i^{\prime }j^{\prime }}\geq D_{1}, \end{array}$$

where *W*_*E*_ = 1.6 and *W*_*I*_ = 2.1 are the total excitatory and inhibitory coupling strengths, respectively.

All synaptic connections between neurons are subject to both long-term and short-term plasticity; for long-term plasticity, we incorporate STDP for all synapses as follows:

(5) $$\Delta W_{ij,i^{\prime }j^{\prime }}\left(t\right)=\left\{ \begin{array}{l} \sum_{t_{ij}^{k}<t}\sum_{t_{i^{\prime }j^{\prime }}^{l}<t}H\left(t_{ij}^{k},t_{i^{\prime }j^{\prime }}^{l}\right)\text{ if }d_{ij,i^{\prime }j^{\prime }}\leq D_{1},\\ 0\text{ if }d_{ij,i^{\prime }j^{\prime }}>D_{1}, \end{array}\right.$$

where *t*^*k*^_*ij*_ is the *k*th firing time of neuron (*i, j*) and *t*^*l*^_*i*′*j*′_ is the *i*th firing time of neuron (*i*′, *j*′), the sum is over all spike pairs before time *t*, and *H(t^k^_ij_, t^l^_i′j′_)* is the STDP window function:

(6) $$H\left(t_{post},t_{pre}\right)=\{\begin{array}{ll} A_{+}\exp\left(-\Delta t/\tau_{+}\right) & \text{if }\Delta t>0,\\ -A_{-}\exp\left(\Delta t/\tau_{-}\right) & \text{if }\Delta t<0,\\ 0 & \text{if }\Delta t=0, \end{array}$$

where *t*_*pre*_ and *t*_*post*_ are the firing times of the pre- and postsynaptic neurons, respectively, and Δ*t* = *t*_*post*_ − *t*_*pre*_. The STDP timescales, τ_+_ = τ_−_ = 20 ms, are similar to values found in experimental studies (Bi and Poo, 1998; Zhang et al., 1998), and *A*_+_ = *A*_−_ = 0.00025, making *H* an odd function in Δ*t*. We incorporate the STDP into the coupling strength to obtain the time-dependent coupling strength (Palmer and Gong, 2013):

(7) $$W_{ij,i^{\prime }j^{\prime }}\left(t\right)=W_{ij,i^{\prime }j^{\prime }}+\Delta W_{ij,i^{\prime }j^{\prime }}\left(t\right).$$

STDP may cause some coupling strengths to reach values that are not within biological limits; to prevent this, STDP requires a hard limit of synaptic modification (Song et al., 2000; Izhikevich et al., 2004). No further constraints are required. We limit changes to coupling strengths as follows:

(8) $$\begin{array}{l} \left|(1-\Delta W_{max})W_{ij,i^{\prime }j^{\prime }}\left(0\right)\right|\leq \left|W_{ij,i^{\prime }j^{\prime }}\left(t\right)\right|\\ \;\leq \left|(1+\Delta W_{max})W_{ij,i^{\prime }j^{\prime }}\left(0\right)\right|, \end{array}$$

where *W_ij,i′j′_*(0) is equal to the initial connection strengths as described in Equation (4), and *W*_*max*_ = 0.18 controls the maximum possible amount of STDP. The results discussed later are not sensitive to this value.

Short term plasticity, also called dynamical synapses, refers to a phenomenon in which synaptic efficacy changes over time such that it reflects the spiking history of a neuron. In contrast with long-term plasticity dynamics such as STDP, short term plasticity induces temporary modification to synaptic efficacy; that is, without continued presynaptic activity, the synaptic efficacy will return to its baseline level. We incorporate such short term plasticity by using the model described in Markram et al. (1998); in this model the synaptic efficacy *A^k^_i′j′_* of the neuron at (*i′, j′*) is

(9) $$A_{i^{\prime }j^{\prime }}^{k}=2u_{i^{\prime }j^{\prime }}^{k}r_{i^{\prime }j^{\prime }}^{k},$$

(10) $$u_{i^{\prime }j^{\prime }}^{k}=U+u_{i^{\prime }j^{\prime }}^{k-1}\left(1-U\right)\exp\left(-\Delta_{i^{\prime }j^{\prime }}^{k-1}/F\right),$$

(11) $$r_{i^{\prime }j^{\prime }}^{k}=1+\left(r_{i^{\prime }j^{\prime }}^{k-1}-r_{i^{\prime }j^{\prime }}^{k-1}u_{i^{\prime }j^{\prime }}^{k-1}-1\right)\exp\left(-\Delta_{i^{\prime }j^{\prime }}^{k-1}/D\right),$$

(12) $$u_{i^{\prime }j^{\prime }}^{1}\;=U,r_{i^{\prime }j^{\prime }}^{1}=1$$

where *A*^*k*^_*i*′*j*′_ represents the absolute synaptic efficacy of the *k*th spike of the neuron, *u*^*k*^_*i*′*j*′_ represents the utilization of synaptic efficiency, *r^k^_i′j′_* represents the availability of synaptic efficiency, Δ^*k*−1^_*i*′*j*′_ is the time between the *k* −1 th and *k*th spike of the neuron located at (*i′, j′*) and *F* = 0.005 *s*, *D* = 0.11 *s* are the timescales representing the recovery of the *u* and *r* variables, respectively (Markram et al., 1998). Since *D* » *F*, these synapses are depressed, i.e., the amplitudes of excitatory postsynaptic potentials caused by closely-spaced successive spikes decrease over time; this is referred to as short term depression (STD). Furthermore, the closer in time successive spikes are, the smaller the efficacy of the spikes, with synaptic efficacy recovering over a timescale which is ≈*D*. Combining STDP described in Equation 7 with STD described in Equations 9–12, the input the neuron (*i, j*) receives from other neurons to which it is coupled is:

(13) $$V_{ij}^{in}\left(t\right)=\sum_{i^{\prime }j^{\prime }}W_{ij,i^{\prime }j^{\prime }}\left(t\right)A_{i^{\prime }j^{\prime }}^{k}\Theta \left(V_{i^{\prime }j^{\prime }}-V_{th}\right),$$

where Θ(*x*) is the Heaviside step function: Θ (*x*) = 1 if *x* ≥ 0, and Θ (*x*) = 0 if *x* < 0.

Since real neural systems are full of noise, we also add noise to the model. We incorporate noise through adding noisy spontaneous firing; for each neuron, a spike is emitted with small probability to generate random sparse spontaneous firing with a frequency around 0.1 spikes per neuron per second, which is within the range of sparse spontaneous firing rate as found in cortical neurons (Griffith and Horn, 1966; Koch and Fuster, 1989).

## Results

### Associative learning based on the interactions of spike waves

**Figure 1:**
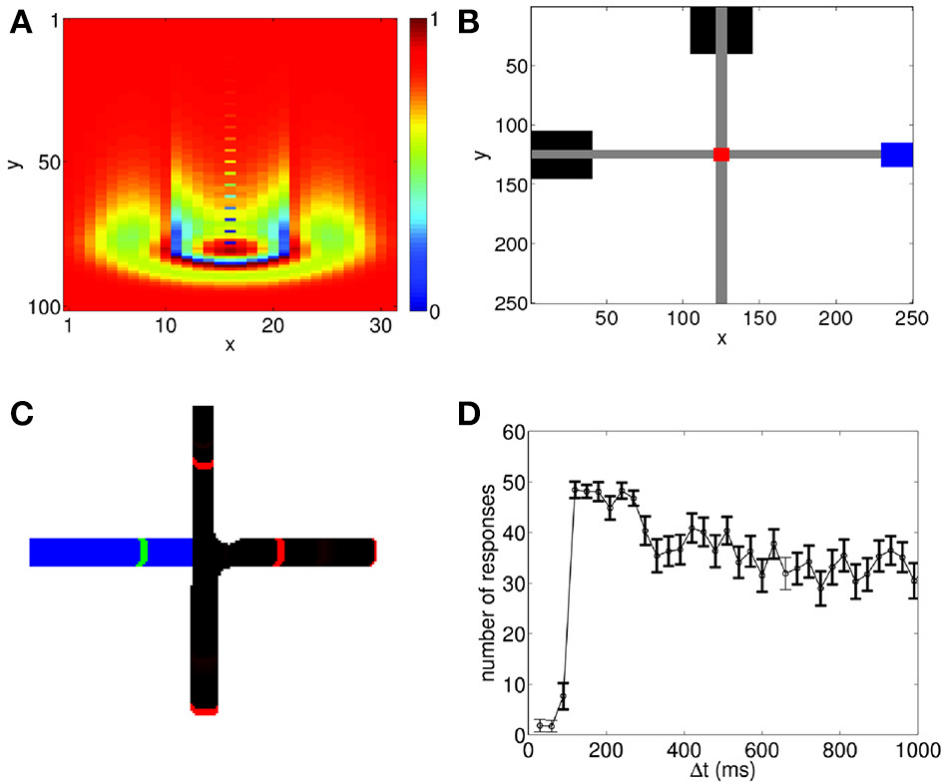
**Propagating spiking waves in the neural circuit model can be used to implement associative learning. (A)** A wave propagating through the neural circuit with *V*^*ex*^ = 0.0429. Color shows the value of *V_ij_(t)*. The wavefront and its tail with reduced potential are clearly visible. **(B)** Schematic of wave interactions for associative learning. Black boxes show the location of the stimuli where the CS is initialized at the top of the network and the US on the left. Gray regions indicate the paths of the propagating spiking waves, the red region shows the interaction region, and the blue region shows the response area. **(C)** Schematic of wave regeneration. The wave encoding the CS (red wavefronts, black path) is initialized at the top of the network and begins propagating toward the interaction region. When it enters the interaction region, it regenerates the US (green wavefront, blue path), which was initialized at the left of the network, thereby achieving associative learning. **(D)** Number of successful associations in 60 trials. There is a sharp increase in the number of successful associations with a peak near 100 ms, followed by a gradual decline. To ensure that the predictive relationship is maintained, we test to see if the US can regenerate the propagating wave encoding the CS. Bolded points show when the number of responses in this case is significantly lower (*p* < 0.01 using a *t*-test) than the number of successful associations; this indicates that the predictive relationship between the CS and the US has been maintained.

To model the association of the conditioned and unconditioned stimuli, we first demonstrate how each stimulus can be represented in the network. To this end, we add a brief localized stimulus representing either the CS or the US to neurons at different locations in the network. These stimuli are able to evoke spiking wave patterns as shown in Figure 1A. As the wave pattern propagates across the network, it naturally gives rise to a spike timing sequence; each stimulus, therefore, is encoded by a spike sequence. In the present study, we use the terms spike sequence and propagating spiking wave interchangeably. This method of generating spike sequences utilizing propagating patterns in recurrent neural circuits is similar to that previously used in Itskov et al. (2011), in which spike sequences formed by localized moving patterns were used to encode elapsed time in behaviorally relevant contexts. Representing external stimuli utilizing spike sequences in our model is consistent with a growing body of experimental results showing that such sequences pay an important role in a host of perceptual and cognitive processes (Pastalkova et al., 2008; Bathellier et al., 2012; Harvey et al., 2012; Xu et al., 2012).

As shown in Figure 1B, both the CS and the US can evoke propagating waves which naturally produce spike sequences; in this example, the CS is represented by the spiking wave traveling from top to bottom, and the US is represented by the spiking wave traveling from left to right. Here we assume that when the wave evoked by the US reaches the corresponding blue box on the right of Figure 1B, a response corresponding to the US is generated; we do not explicitly model how the direction of wave propagation can be read out to generate a behavioral response. However, such read out could be achieved through spatial arrangement of dendritic receptor fields. Indeed, it has been found that dendrites are sensitive to spike sequences arriving from different directions (Branco et al., 2010); in a recent modeling study, this property has been used to read out information contained in propagating waves (Heitmann et al., 2013).

We now demonstrate that associative learning of the CS and the US naturally arises in the network with STDP and STD. Although there has been a significant number of experimental studies measuring how such learning can occur in a variety of situations, the general protocol that has been used is: the two paired stimuli are presented repeatedly to allow learning to happen; the CS is presented first. After a delay time, Δ*t*, which is constant for all learning trials, the US is then presented. After training, when the CS is presented, the response of the US is generated, indicating that the CS can be taken as a predictor for the US (Rescorla, 1988). Using the same protocol as used in behavioral studies, we apply the paired stimuli to the network model for 60 trials. After some learning trials (typically < 20), when the CS is presented alone, it is able to generate the unconditioned response. As illustrated in Figure 1C, this occurs because when the spike sequence (wave) encoding the CS propagates along its path, it is able to regenerate the spike sequence (wave) that encodes the US.

### The temporal contiguity property of conditioned associative learning

As shown in behavioral studies, the success of associative learning of conditioning is a non-monotonic function of the amount of time, Δ*t*, by which the CS precedes the US (Rescorla, 1988). When Δ*t* is either too short or too long, conditioning is relatively poor. However, there is some intermediate set of time intervals when conditioning is most successful. This temporal contiguity property of classical conditioning has been found to be invariant across many different experiments covering a wide variety of stimuli, although there is no absolute temporal interval that produces the best conditioning. For instance, a similar non-monotonic functional form has been found when testing both rabbit eyelid response and pigeon keypeck response, however, the time intervals for optimal conditioning were on the order of 100 ms and 5 s, respectively (Smith et al., 1969; Barker and Smith, 1974; Rescorla, 1988).

We now demonstrate that this temporal contiguity property can be reproduced by our propagating wave-based model of associative learning. When both STDP and STD are included, we run 60 trials of the paired US and CS. As shown in Figure 1C, for some values of Δ*t*, after training it is possible for the CS to generate the response of the US. In our testing regime, we apply the CS alone after each learning trial. If the US response is generated, it is counted as a successful association. As in behavioral studies (Rescorla, 1988), we count the number of successful trials, and repeat this procedure for different Δ*t* values to study how successful conditioning varies with Δ*t*. Figure 1D shows that our model is able to reproduce the temporal contiguity property of conditioning, as observed in experimental studies (Smith et al., 1969; Barker and Smith, 1974; Figure 1 of Rescorla, 1988). In particular, the model shows a very low number of responses for small Δ*t* followed by a sharp increase with a peak at Δ*t* ≈ 100 ms, which is then followed by a decay in number of responses for larger Δ*t*. The associative learning therefore is relatively poor if Δ*t* is either too short or too long, and it appears to be a non-monotonic function of Δ*t*. Note that despite the general trend of the decay of successful learning for larger Δ*t* (the mechanism underlying such decay is illustrated below), a significant number of successful learning can still happen even when Δ*t* ≈ 1 s, a time scale that is much larger than that of individual neurons or synapses.

### Mechanisms of associative learning based on spiking wave interactions

As demonstrated above, associative learning of the CS and the US can naturally occur in our model, utilizing spike sequences generated in the network. The question that arises naturally is: what are the mechanisms underlying such associative learning? It has been proposed that spike sequences are important neural substrates for perceptual and cognitive functions (Abeles, 1991; Kumar et al., 2010). However, the timing relationship between such sequences has only been explored for their zero-lagged synchrony and its potential computational roles for feature binding (Abeles et al., 2004). As proposed recently, cell assemblies organized as spike sequences could be the basic tokens of the “neural syntax” (Buzsáki, 2010), just as words are the basic tokens of the syntax of language; clearly, such complex neural syntax requires that different spike sequences exhibit timing relationships more complicated than those of zero-lagged synchrony. We now demonstrate that the spike sequences formed in our neural circuits have complex interactions that are timing dependent and that such timing-dependent interactions of spike sequences are essential for implementing associative learning at the level of neural circuits.

**Figure 2:**
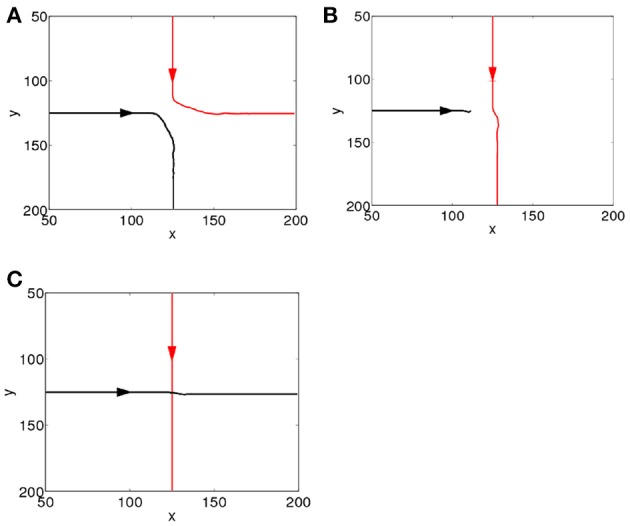
**Interacting dynamics of spiking waves**. The propagating path of the wave encoding the CS is denoted by the red line; that of the wave encoding the US is denoted by the black line. **(A)** A repulsive interaction (Δ*t* = 0 ms). **(B)** A suppressive interaction (Δ*t* = 20 ms). **(C)** The second wave passes through the tail of the first (Δ*t* = 360 ms).

To study how the interactions between the propagating spiking waves evoked by the CS and the US depend on their time separation, Δ*t*, we systematically vary Δ*t* from 0 to 1000 ms. Since the CS is added to the network prior to the US, for clarity we label the spiking waves evoked by the CS and US as the first and second waves, respectively. We first investigate the behavior of the network in the first learning trial as Δ*W_ij,i′j′_(t)* in Equation 5 is small, and the additional dynamics added by STDP are minimal, which simplifies analysis. As shown in Figure 2, the interactions can be largely grouped into three main categories, namely, repulsion, suppression and pass-through. The repulsive interaction occurs when the second wave interacts with either the front or the tail of the first wave, causing the paths of the both spike waves to be altered; this typically occurs for small values of Δ*t* (Δ*t* <15 ms). For larger values of Δ*t* (15 < Δ*t* < 100 ms), suppression can occur when the second wave interacts with the tail of the first one, as a result the second wave is annihilated. For both the repulsive and suppressive interactions, it is apparent that the wave evoked by the US is unable to pass through the path of the one evoked by the CS; i.e., the propagation path of the first wave is blocked by the tail of the second wave. However, when Δ*t* > 100 ms, the second wave is able to pass through the path of the first one. These wave interactions therefore depend on their temporal separation; in other words, their interaction dynamics are timing dependent. Similar timing-dependent interaction dynamics of evoked propagating waves has been found in the rat visual cortex; as reported in Gao et al. (2012), when the inter-stimulus interval was between 80 and 300 ms, a second propagating wave was significantly suppressed by the first one. This is similar to the suppressive interactions shown in our model and there is certain overlap between the interval (80–300 ms) found in Gao et al. (2012) and that in our model (15 – 100 ms). For inter-stimulus intervals greater than 300 ms, the amplitudes of the two waves showed no significant difference (Gao et al., 2012). This is similar to the pass-through that we observed for large values of Δ*t*, although for our model this behavior occurs when Δ*t* > 100 ms.

### The roles of STDP and STD in associative learning

In the previous Section, we have illustrated the dynamics of interactions between multiple propagating spiking waves when Δ*W_ij,i′j′_(t)* is small (i.e., the beginning of the learning process). After allowing learning to proceed, when Δ*t* is small, the blocking effect still exists as shown in Figures 2A,B. When Δ*t* is large, the second wave can freely pass through the tail of the first one (Figure 2C); however, after several trials of learning, the presence of the first wave along can regenerate the second one, as shown in Figure 1C. We now show that this occurs because STDP plays a crucial role in learning the paths of the propagating waves and their interactions.

**Figure 3:**
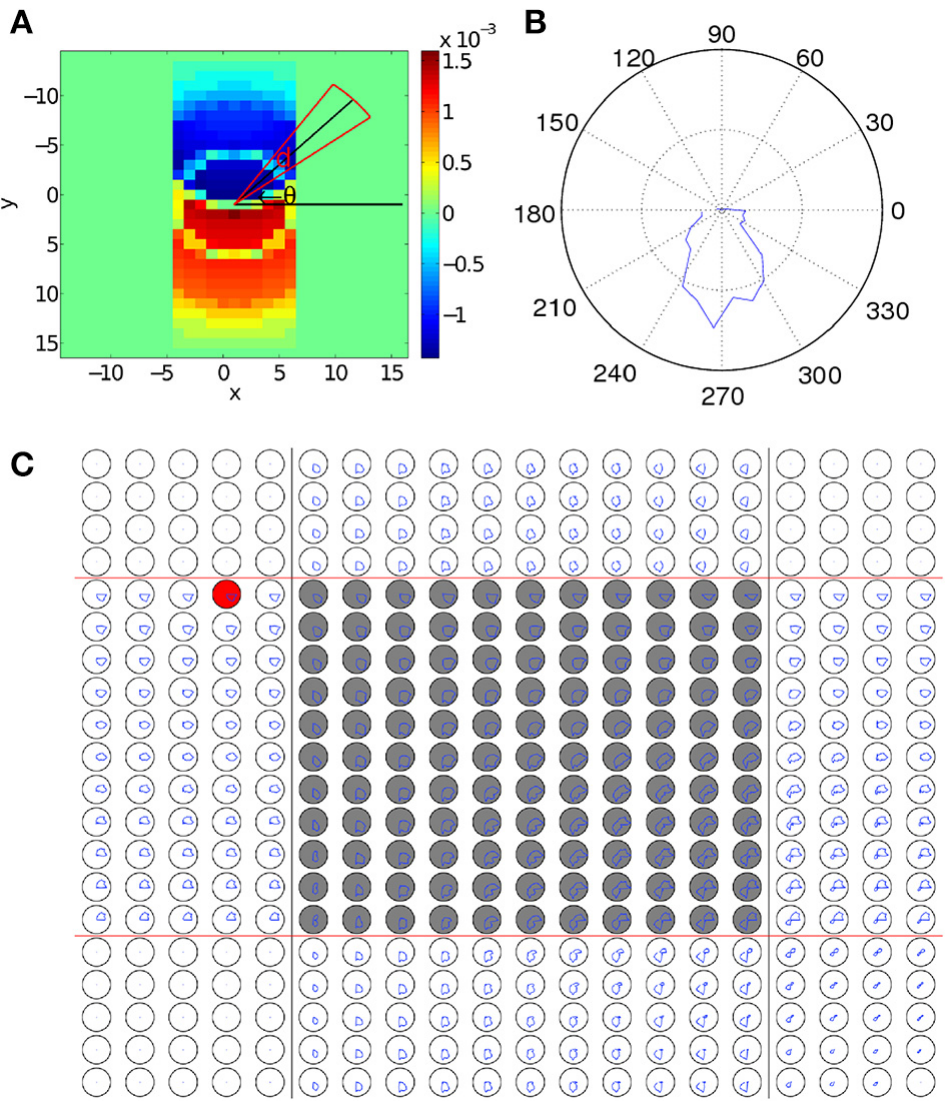
**Changes to synaptic coupling strengths due to STDP. (A)** Changes in coupling strength (Δ*W_ij_,i′j′(t)*) after a wave pattern has traveled over a neuron. Coupling strengths in the direction of propagation are increased, and strengths in the opposite direction are decreased. To calculate Λ_*ij*_ (θ), the coupling strength changes within the circular sector (red lines) centered at an angle of θ (black lines) are averaged to give the value of Λ_*ij*_ (θ). **(B)** Λ_*ij*_(θ) for the neuron in **(A)**. There is a clear peak pointing in the direction of wave propagation across the neuron. **(C)** Λ_*ij*_(θ) for the 20 × 20 sub-grid of neurons near the interaction region. The original paths of the two wave patterns corresponding to the CS and the US are marked with black and red lines, respectively. The gray-filled circles mark neurons in the interaction region. The polar plots are laid out so that each plot corresponds directly to a single neuron in the grid of neurons. For scale, the black circles show a mean change in connection strength of 10^−4^. In the left and top of the figure, only a single wave passes over each neuron, leading to connection increases in only one direction, similar to **(B)**. The areas in the corners with no connection strength changes because in the regions the wave does not pass over. In the interaction region, neurons typically show an increase in coupling strength in two directions, corresponding to the top-to-bottom CS and the left-to-right US. The resulting bimodal distribution of coupling strengths enables the spontaneous wave regeneration to happen. The red-filled circle shows a neuron on the edge of the path. As Λ_*ij*_(θ) is slightly biased toward the center of the wave pattern, waves initialized near this neuron will quickly converge to the learned path.

We first examine how coupling strengths are changed as a result of STDP during the learning process of the paired CS and US. As a wave pattern propagates across the neural circuit, the coupling strengths aligned with the direction of its propagation will be increased as STDP increases coupling strengths when presynaptic neurons fire before postsynaptic ones. On the other hand, as the STDP window function (Equation 6) is odd, connections in the direction opposite to wave propagation will be decreased, as shown in Figure 3A. Whilst coupling strength changes resulting from a wave pattern traveling over a path once are relatively small, if it repeatedly travels over the same path (as in the training process) significant changes to coupling strengths can occur, with the maximum change limited by the bounds to STDP (Equation 8). As shown in Figure 3A, it is simple to understand coupling strength changes due to a single pattern, but analyzing the changes to connection strengths within the interaction region are more complex, as each neuron has around 700 connections and multiple wave patterns travel over each neuron from different directions. To simplify, we define a function that can be used to quantify changes to the strength of synaptic connections from a single neuron as a function of a single variable, namely the angle of the connection on the two-dimensional, spatially extended spiking neural circuit. This is achieved by creating a new polar coordinate system centered on the neuron of interest and averaging connection strengths over certain angular segments (Figures 3A,B). The change of coupling strength as a function of angle is:

(14) $$\Lambda_{ij}\left(\theta \right)=max\left(\frac{\begin{array}{c} \sum_{x=-D1}^{D_{1}}\sum_{y=-D1}^{D_{1}}\Delta W_{ij,i+x\;y+j}\left(t\right)\\ \Theta \left(d-\left|\theta -\tan^{-1}\left(\frac{x}{y}\right)\right|\right) \end{array}}{\begin{array}{c} \sum_{x=-D1}^{D_{1}}\sum_{y=-D1}^{D_{1}}\\ \Theta \left(d-\left|\theta -\tan^{-1}\left(\frac{x}{y}\right)\right|\right)\; \end{array}},0\right),$$

where *d* is a small number that controls the angular resolution of Λ_*ij*_(θ), *D*_1_ is the maximum coupling distance (Equation 2), and *x* and *y* index over all neurons that the neuron at location (*i, j*) is connected to. Λ_*ij*_(θ), defined in Equation 14, measures the amount of synaptic changes caused by STDP as a function of angle, θ, from the presynaptic neuron (Figure 3A). This is calculated by summing over all connected neurons and then using a Heaviside step function to ignore those neurons which are connected to the main neuron at angles different from θ, i.e., neurons outside the red sector in Figure 3A. As neurons are aligned along a regular grid, the spatial density of connections is uniform but the angular density of connections is non-uniform; to correct for this, we normalize Λ_*ij*_(θ) by dividing it by the number of neurons that we are averaging over. The negative component of Δ*W_ij,i′j′_* is removed for simplicity, as it is almost always in the exact reverse direction when compared to the positive component.

As discussed above, we expect that when a single wave passes over a neuron, Λ_*ij*_(θ) will have a single peak that is approximately aligned with the direction of propagation of the wave. This is shown in Figure 3B where Λ_*ij*_(θ) shows a single peak, which is aligned with the propagation direction of the wave. We now calculate Λ_*ij*_(θ) for all neurons near the interaction region after training by repeatedly applying the paired US and CS stimuli, as shown in Figure 3C. We find that in this case, the behavior of Λ_*ij*_(θ) near the interaction region is more complex than the case when only a single wave is present (Figure 3B). In particular, as waves are interacting within this region, both wave patterns cause changes to coupling strengths in their directions of propagation. As a result, neurons within the interaction region show two peaks in Λ_*ij*_(θ), corresponding to the directions of the two propagating wave patterns (Figure 3C), namely, top-to-bottom and left-to-right. This bimodal distribution of coupling strengths developed during the learning process endows the network with the property of spiking wave pattern regeneration as shown in Figure 1C. Specifically, when the wave encoding the CS propagates alone and enters the interaction region (Figure 3C), neurons to the right of the wave pattern will receive significant excitatory input due to the increased coupling strengths in that direction (Figure 3C). This will cause certain neurons in that direction to fire; if the number of these firing neurons reaches a critical value, it is possible for a wave pattern propagating along the right-to-left direction to be regenerated and this regenerated wave will then travel along the path of the wave evoked by the US. This wave regeneration allows for the wave evoked by the CS to generate the unconditioned response, a key feature in our associative memory model (Figure 1C). Here it is interesting to note that in our theoretical account of associative learning, the neurons in the intersection region play an essential role in associating the US and the CS together; this notion is similar to the concept of “nodal” neurons discussed in Eichenbaum et al. (1999), in which it was hypothesized that some nodal neurons could encode the common features of different temporal events, so that these temporal events could be associated to form episodic memory (see Figure 3 in Eichenbaum et al., 1999).

Aside from changing coupling strengths during the training process (Figure 3), STDP also has a notable secondary effect, namely, that the speed of spiking wave propagation undergoes an increase of 25% after only 5 training trials. This property is qualitatively similar to the experimental data reported in Xu et al. (2012), in which visual stimulus (a moving dot) could induce spike sequences and the propagation speed of these sequences increased after a training process, i.e., many repeated presentations of the stimulus. Such increased speed of training or experience-induced spike sequences has also been observed in the hippocampus and in the neocortex (Ji and Wilson, 2006; Euston et al., 2007).

**Figure 4:**
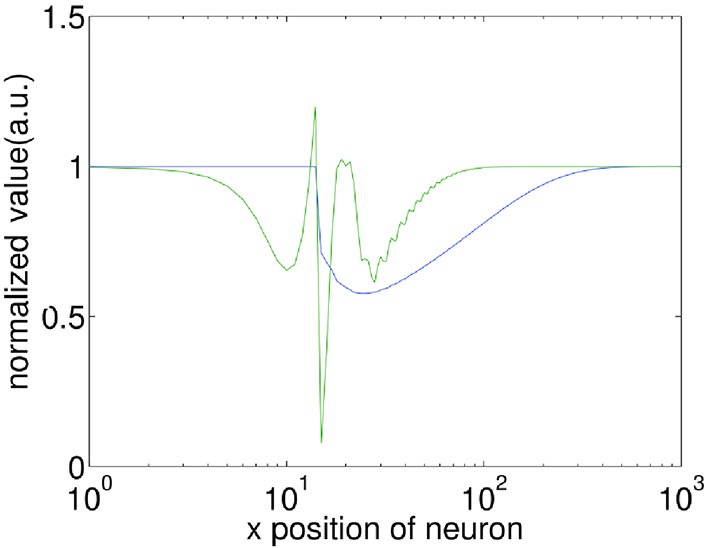
**Comparison of the recovery process of the membrane potential (*V*) and synaptic efficacy (*A*)**. A propagating spiking wave is initialized and the value of *V* and *A* is measured along its path. Both are normalized with their resting values equal to 1 enabling them to be plotted on the same scale for comparison. Note that the x-axis is a log scale, whilst the y-axis is linear. The front of the wave is near *x* = 10, at the peak of the green line which indicates a neuron emitting a spike. It is clear that the membrane potential has recovered to close to the initial value within ≈100 grid units of the wavefront, whilst the synaptic efficacy takes ≈500 grid units to recover.

In associative learning of classical conditioning, there is a predictive relationship between the CS and the US. This allows the CS to predict the US, whilst the US does not predict the CS (Rescorla, 1988). In our model, we find that with STDP but without STD, this predictive relationship is violated when Δ*t* » 200 ms, as the second wave encoding the US is capable of generating the CS response. This happens because without STD, when the second wave approaches the interaction region, the membrane potential of neurons along the paths of the first and the second wave has been restored to their initial values, thus the membrane potential would be near-identical for both the first and the second waves. Incorporating STD, however, breaks this symmetry by adding an extra slowly-recovering effect, namely synaptic efficacy. As shown in Figure 4, the reduction in synaptic efficacy caused by the propagation of the first wave persists long after the neuron membrane potentials have recovered to their equilibrium values. As described in Equation 13, synaptic efficacy can affect the total inputs to neurons; namely, the smaller synaptic efficacy is, the smaller the inputs to other neurons are. This means that when the second wave reaches the interaction region (Figure 3C), the inputs caused by its spiking wave front to the neurons along the direction of the first wave are reduced due to the small synaptic efficacy caused by the first spiking wave. In this way the second wave is unable to regenerate the first wave; therefore, the predictive relationship between the CS and the US is maintained.

### Decay of successful association due to noisy spontaneous firing activity

The general function form of the temporal contiguity property of classical conditioning is that the success of association between the CS and the US is a non-monotonic function of their time separation, Δ*t*, as found in experiments (Rescorla, 1988) and reproduced in our modeling study (Figure 1D). In particular, for large values of Δ*t*, the amount of successful association decreases as Δ*t* increases; this decay of association over time is ubiquitous for different experimental protocols (see Figure 1 of Rescorla, 1988), but the underlying neural mechanism still remains unclear. We now show that in our model, this decay of association occurs due to the random modifications of synaptic coupling strengths caused by spontaneous firing activity (see Materials and Methods).

**Figure 5:**
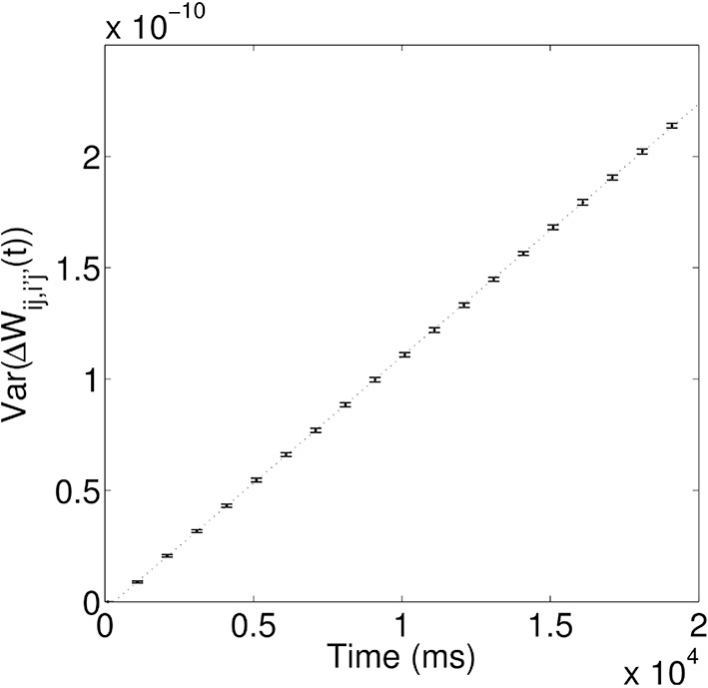
**Variance of random synaptic modifications over time**. This variance increases linearly over time, i.e., σ^2^(*t*) ∝ τ, indicating Δ*W_ij,i′j′(t)_* undergoes a random walk. For the linear fit, the coefficient of determination, *R*^2^ = 0.99997.

As demonstrated above, during the training process, the synaptic coupling strengths along the propagation paths of the first and second waves are modified by STDP to store these spiking waves. However, due to the random nature of the spontaneous firing activity, these coupling strengths will be changed in a random way; i.e., the coupling strengths will be randomly potentiated or depressed through STDP, depending on the temporal order of the random spikes happening to two coupled neurons. One would then expect that such random changes can make the coupling strengths to behave like a random walk. To test this, we calculate the variance of the random change of coupling strengths Δ*W_ij,i′j′_(t)* as a function of time. Figure 5 shows that that the variance increases linearly over time, i.e., σ^2^(*t*) ∝ *t*, which is a characteristic feature of a random walk. As in Fusi and Abbott (2007), we then define the memory traces of the propagating spiking waves stored in the modified synapses as “signal,” *S*. The random walk of coupling strengths due to random spontaneous firing activity would cause the memory traces to fluctuate and degrade; such fluctuation introduces “noise,” *N* to the memory traces, defined as the standard deviation of the random changes to the signal. After the training process, the long-term modifications of synaptic strengths due to the propagating waves are Δ*W_ij,i′j′_*, so the signal *S* ∝ Δ*W_ij,i′j′_*. Due to its random walk nature, the random modification of synaptic strengths *N* ∝ *t*^1^/2. The quantity that can be used to characterize the quality of the stored memory of propagating waves is the signal-to-noise ratio, *S*/*N*, and *S*/*N* ∝ Δ*W_ij,i′j′_/t*^1^/2. The signal-to-noise ratio thus decays monotonically as Δ*t* increases, meaning the memory traces of propagating waves decay monotonically; this therefore results in a reduction in the number of successful associations between the two stimuli.

### Robustness of associative learning

**Figure 6:**
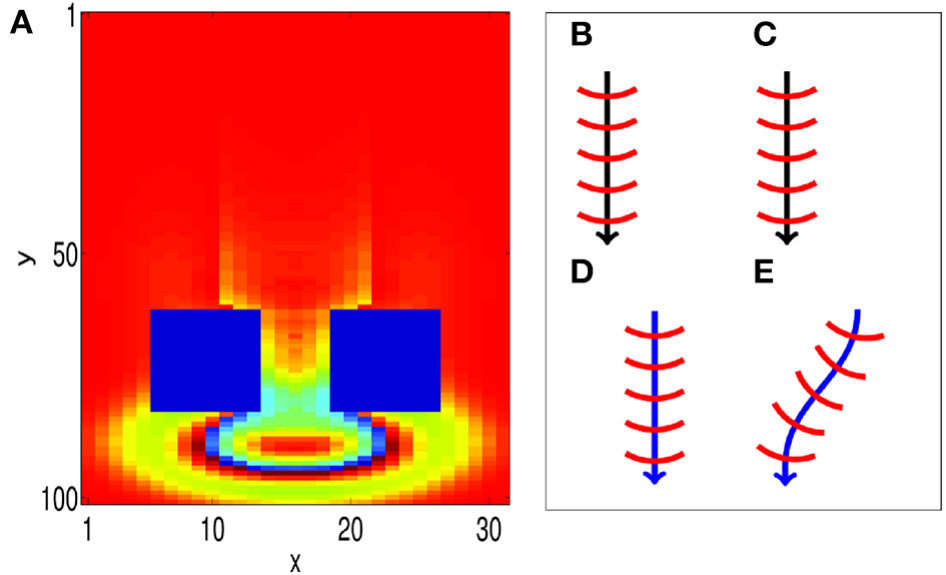
**Propagating spiking waves are stable with respect to perturbations. (A)** The size of traveling wave patterns is stable. We initialize a wave pattern at the top of the image. It then propagates toward two regions that are fixed with *V_ij_(t)* = 0 (the two blue rectangles). After traveling past these obstacles, the wave pattern quickly returns to its original size. This demonstrates that the size of the wave pattern is stable. **(B–E)** STDP can enable a path to be learned (schematic). **(B,C)** show initial training of a path without and with STDP, respectively. **(D,E)** Show the behavior when a test pattern is initialized to the side of the original pattern. With STDP **(E)**, the pattern converges to the original path. Without STDP **(D)**, the pattern continues in a straight line.

We now show that our associative learning implementation is robust to external perturbations. The propagating waves used to encode the CS and the US have wavefronts that are composed of many firing neurons, which make them robust to some perturbations. In particular, when a propagating wave front is only partially activated by external perturbations, it can recover to its original size; this is illustrated in Figure 6A, where an obstruction is created by forcing blocks of neurons to zero potential, i.e., *V_ij_(t)* = 0. After propagating through the obstacle, the wave pattern quickly returns to its original size, before continuing to propagate along its original path. The robustness of the size of the wave pattern can be understood through the shape of the coupling function (Equation 2). We note that the wave pattern size is close to the minimum distance for inhibitory connections *D*_0_. As a result, if the wave pattern expands, the amount of inhibition increases, allowing the pattern to return to its original size. Similarly, if the pattern shrinks, the amount of inhibition decreases, allowing the pattern to return to its original size.

Furthermore, the model is robust with respect to perturbations in the CS and US locations. We consider two cases; one with STDP and another without STDP to illustrate the importance of STDP in correcting such perturbations. We first run training, as shown in Figures 6B,C, by presenting a stimulus at the same location for both cases. After training, the location of the wave initialization site is shifted slightly to a different side to test whether it can return to the learned path, as shown in Figures 6D,E. When STDP is included in the network (Figure 6E), the test pattern quickly returns to the original learned path, indicating that for this case, the learned spiking wave is robust to the changes of the initial stimulus location. Such perturbations in the initial locations can be regarded as errors in the initial stimulus. The dynamical behavior of returning to the learned path therefore indicates that the dynamics of the wave patterns now have an error-correcting property. This error-correcting property is analogous to that shown in conventional attractor neural networks (Hopfield, 1982), in which perturbations to state away from a learned attractor are corrected as the state quickly returns to the attractor. To understand how STDP has enabled such error correction, we investigate how STDP has affected the coupling strengths of neurons as waves propagate across them, as shown in Figure 3C; in particular, the red-filled circle in the figure represents a single neuron located near the edge of a wave pattern. We observe that Λ_*ij*_(θ) is angled toward the center of the wave pattern. As a result of these modified coupling strengths, neurons on the original path receive extra excitation. This causes the wave to return to the learned path, as shown in Figure 6E. However, without STDP, the test pattern does not return to the previous path; instead, it propagates along a different path (Figure 6D).

## Discussion

In this study, we have proposed a novel theoretical account of associative learning of temporally disparate events in the context of classical conditioning. In our model, temporally separated events such as the CS and the US are encoded by different spike sequences formed from propagating spike waves. After training, the sequence encoding the CS can regenerate the sequence encoding the US, therefore enabling successful association between them. Associative learning therefore happens in a distributed way at the level of neural circuits, without slowly decaying firing activity of neurons. Furthermore, as we have demonstrated, our model is able to account for the temporal contiguity property of classical conditioning and is robust to certain types of noise perturbations.

In our model of associative learning, the timing relationships of firing activity of groups of neurons, including spike sequences formed from these neurons and their timing-dependent interactions are essential for associative learning. Spike sequences have indeed been found to be important for a range of perceptual and cognitive processes (Pastalkova et al., 2008; Bathellier et al., 2012; Harvey et al., 2012; Xu et al., 2012). However, most of these studies have focused on just one such sequence and higher-level timing relationships between different sequences have not been explored. Similarly, previous modeling studied have mainly focused on modeling the formation of spike sequences (Abeles, 1991; Kumar et al., 2010); interactions between different sequences have been rarely discussed and such discussion has just focused on their zero-lag synchrony and its role in feature binding (Abeles et al., 2004). However, rich timing-dependent interactions of different spike sequences, as illustrated in our model in the context of associative learning, could be of fundamental importance for brain functions.

In our model of associative learning of classical conditioning, propagation of spiking waves across neural circuits is the mechanism underlying the formation of spike sequences. As we have illustrated, such propagation of neural activity is essential to make local information available at larger spatial and temporal scales, therefore associating signals that are distributed over space and time. Interestingly, the notion that neural activity propagation is important for classical conditioning is reminiscent of Pavlov's original speculation that “automatic irradiation” of neural firing activity evoked by external stimuli could be the neural mechanism underlying classical conditioning (Pavlov, 1927). Recently, evidence of propagating waves in the brain has been accumulating very rapidly (Rubino et al., 2006; Benucci et al., 2007; Ferezou et al., 2007; Wu et al., 2008; Lubenov and Siapas, 2009; Sato et al., 2012). In Ferezou et al. (2007), it was speculated that multiple waves activated in a distributed manner may be essential for “associative plasticity and learning.” In Gong and Van Leeuwen (2009), it was proposed that propagating wave patterns are basic building blocks for neural circuits to carry out distributed dynamic computation; in this paradigm, information is encoded in these dynamic patterns and communicated to the different parts of the cortex based on their propagation, and information is processed when they collide or interact with each other. Here, we have presented such a model for associative learning based on interacting spiking wave patterns.

Interacting waves have been used to account for classical conditioning in Beurle (1956), in which it was shown that the CS and the US can evoke two plane waves. After learning, which was implemented through modulating firing thresholds of neurons, the activation of one wave is able to regenerate another wave, referred to as “pulse regeneration” (Beurle, 1956). In this way, an association between two events can be formed. In Beurle's study, two waves which occurred at the same time were used to model association between the CS and the US; this is in contrast to real psychophysical data showing that when the CS and the US occur together, no association can be formed (Rescorla, 1988). In our study, however, the temporal separation between the US and the CS is explicitly modeled and a salient feature of classical conditioning, namely the temporal contiguity of classical conditioning can be reproduced. Furthermore, in our model, the learning of wave propagation paths is achieved by using synaptic dynamics such as STDP and STD that have been widely observed in many different parts of the brain (Abbott and Nelson, 2000; Chung et al., 2002); in Beurle's original work, however, there was no explicit synaptic plasticity considered and experimental evidence of learning achieved by modulating firing threshold is rare.

Both STDP and STD play important roles in our model of associative learning. As we have illustrated, STDP allows spike sequences to be learned and importantly such learned sequences are very robust against different types of perturbations, including changing the location of the initial stimuli. This robust learning of spike sequences can occur because asymmetric synaptic weights developed in our model enable perturbed waves to converge back to a nearby learned path. As we have illustrated, these asymmetric weights develop due to the temporal asymmetry of STDP and because waves move sequentially across the circuit. STDP also pays an important role in associating different events together by increasing the coupling strengths along the propagating paths of different spiking waves to form an intersection point between them. The role of neurons around the interaction points established by STDP in our model of associative learning is similar that of “nodal cells” introduced in Eichenbaum et al. (1999), in which it was proposed that some hippocampal neurons may function like nodal cells to link sequential events to form a memory space.

Short term depression (STD) is another essential element in our model of associative learning. Without STD, the US and the CS would experience the network in an essentially identical state, therefore they are likely to generate similar responses. Previous models have broken this symmetry by assuming the existence of persistent neural firing activity that decays slowly (Drew and Abbott, 2006). However, the existence of such input is still inconclusive (Ito et al., 2008). In contrast, with STD the reduction in synaptic efficacy, after the wave corresponding to the CS propagates through the network, causes the symmetry between the US and the CS to be broken. In this model of associative learning, therefore, there is no need to assume any persistent neural firing activity. Since generating spikes is energetically expensive for neurons (Niven and Laughlin, 2008), our model potentially provides an economical and robust way to implement associative learning.

Since our model for associative learning is based on several general properties of neural circuits, including the existence of stimulus evoked propagating waves, STDP and STD, it may be applicable to a variety of neural systems. Examples to which our model could be applied include the hippocampus, which is generally regarded as a place where association of spatially and temporally disparate events can form (Rawlins and Olton, 1982; Eichenbaum et al., 1999). Notably, both spike sequences and propagating waves such as propagating waves of theta oscillations (Lubenov and Siapas, 2009; Patel et al., 2012) and propagating waves of sharp wave ripples have been observed in the hippocampus (Patel et al., 2013). Another area in neural systems, to which our model can be applied is primary visual cortex, in which it has recently been found that moving stimuli can evoke spike sequences and these sequences can be recalled once a clue is presented (Xu et al., 2012). In these neural systems, it would be interesting to investigate timing-dependent interactions between different spike sequences and their functional roles.

### Conflict of interest statement

The authors declare that the research was conducted in the absence of any commercial or financial relationships that could be construed as a potential conflict of interest.
